# Bacterial Community Patterns Across the Whole-Plant Continuum of *Ormosia microphylla* in Diverse Habitats

**DOI:** 10.3390/microorganisms14051143

**Published:** 2026-05-19

**Authors:** Lixu Li, Feng Chen, Guohua He, Xiao Wei, Feng Wang, Jianmin Tang

**Affiliations:** 1 Guangxi Key Laboratory of Functional Phytochemicals Research and Utilization, Guangxi Institute of Botany, Chinese Academy of Sciences, Guilin 541006, China; lixuli0405@126.com (L.L.); fengchen27@126.com (F.C.);; 2 College of Tourism and Landscape Architecture, Guilin University of Technology, Guilin 541006, China; 3 Deqing Forestry Administration of Guangdong Province, Zhaoqing 526600, China

**Keywords:** endangered plants, germplasm resources, *Ormosia microphylla*, plant conservation, whole-plant continuum bacterial community

## Abstract

*Ormosia microphylla* is a national first-class protected wild plant in China that faces conservation challenges, including weak natural regeneration and limited environmental adaptability. Plant-associated bacterial communities are important components of host-associated microecosystems, but bacterial community patterns across the whole-plant continuum of *O. microphylla* remain poorly understood. To provide a descriptive micro-ecological baseline, we characterized bacterial communities across the rhizosphere–root–stem–leaf continuum of *O. microphylla* in three geographic habitats in Southwest China: karst mountainous area, a plateau-to-plain transitional slope zone, and a hilly area. High-throughput amplicon sequencing was used to analyze bacterial diversity and composition, and co-occurrence network analysis was used to describe statistical associations among bacterial taxa. Three main patterns were observed. First, bacterial alpha diversity generally declined from the rhizosphere to internal tissues (rhizosphere > root > stem > leaf). Second, bacterial composition varied by plant compartment and habitat. Dominant rhizosphere taxa differed among habitats, whereas internal tissues were generally dominated by Proteobacteria. *Delftia* showed relatively high abundance in several endophytic compartments, suggesting that this genus may be considered a candidate endophytic taxon for future validation. Third, co-occurrence network analysis showed habitat- and compartment-associated differences in network size, complexity, and positive/negative co-occurrence patterns. Overall, these results describe compartment- and habitat-associated bacterial community patterns in *O. microphylla* and provide a micro-ecological baseline for future culture-dependent and functional studies.

## 1. Introduction

The genus *Ormosia* (Fabaceae, Papilionoideae), which is mainly distributed in the subtropical regions south of the Wuling Mountains in China, Southeast Asia, northwestern Australia, and tropical America, contains various bioactive components, including alkaloids, flavonoids, and triterpenes. These compounds have antioxidant, anticancer, insecticidal, and antibacterial activities, indicating potential value for medicinal development [1,2]. *Ormosia microphylla* (*O. microphylla*), a member of this genus, is an endemic species in China and has a relatively narrow geographic distribution, occurring mainly in evergreen broad-leaved forests and mixed evergreen–deciduous broad-leaved forests in Guangxi, Guizhou, Fujian, Guangdong, and adjacent regions [3,4]. Owing to its high conservation priority, *O. microphylla* was listed as a national first-class protected wild plant in the newly revised National Key Protected Wild Plants List of China in 2021 [5]. It has also long been valued as a rare woody germplasm resource for furniture, musical instruments and artwork production because of its straight grain, high density, hardness, corrosion resistance and ornamental heartwood. However, wild populations of *O. microphylla* are facing multiple pressures, including historical overexploitation, habitat fragmentation, weak natural reproduction and regeneration, unstable flowering and fruiting, low fruit production, hard seed coats, poor water absorption after drying, irregular germination and strong interspecific competition [6,7,8,9]. In addition, natural habitat changes and anthropogenic activities such as grazing, logging, cultivation and road construction have led to a sharp decline in wild resources [10]. The species is also susceptible to stem borers and pests, such as longicorn beetles (Cerambycidae), with infestation rates exceeding 90% in individuals with a diameter at breast height (DBH) of over 8 cm [11,12]. Current conservation measures, including establishing nature reserves for in situ conservation, germplasm banks, improved seedling management and pest control, are effective to some extent [13,14]. However, these strategies do not directly address the species’ weak natural regeneration or the micro-ecological factors potentially associated with its survival and conservation.

Plant-associated bacterial communities, including rhizosphere, endophytic, and phyllosphere bacteria, are important components of host-associated microecosystems and are closely linked to plant health, nutrient acquisition, and stress responses [15,16]. Rhizosphere bacteria can improve nutrient availability through nitrogen transformation, phosphate solubilization, siderophore production, and organic matter turnover, whereas endophytic and phyllosphere bacteria may contribute to host performance through phytohormone production, 1-aminocyclopropane-1-carboxylate (ACC) deaminase activity, niche occupation, and modulation of host stress responses. Beneficial bacteria may also enhance plant resistance to biotic stresses by suppressing pathogens, competing for ecological niches, producing antimicrobial compounds, and inducing systemic resistance, and they may alleviate abiotic stresses such as drought, nutrient limitation, salinity, and heavy metal stress through multiple physiological and biochemical pathways [15,17,18,19]. These findings indicate that plant-associated bacterial communities have broad functional potential, although their actual ecological roles depend strongly on host species, plant compartment, and environmental context. For endangered plants, plant-associated microbiomes may be particularly relevant because population decline is often accompanied by weak natural regeneration, low seedling establishment, habitat fragmentation, and reduced environmental resilience. Studies on threatened or rare plants have shown that rhizosphere and endophytic microorganisms can be associated with seedling growth, nutrient acquisition, stress tolerance, and disease resistance, and may provide microbial candidates for conservation-oriented propagation or habitat management [20,21,22,23]. For example, a recent study on the endangered Qingdao lily (*Lilium tsingtauense*) showed that microbiome-guided isolation of native rhizosphere fungi identified *Trichoderma longibrachiatum*, which colonized host roots and significantly promoted root growth [24]. These findings suggest that microbiome surveys can serve as an important first step for identifying candidate microbial resources, although their functional roles must be confirmed through culture-dependent isolation and inoculation experiments. However, compared with crop and model plants, microbiome studies on endangered woody plants remain limited, and the spatial organization of bacterial communities across the rhizosphere–root–stem–leaf continuum is still poorly understood.

In this study, we collected whole-plant continuum samples of *O. microphylla*, including rhizosphere soil, roots, stems, and leaves, from three locations: Chazichong (CZC) in Taijiang County, Guizhou Province; Huangjingdong (HJD) in Longlin Various Nationalities Autonomous County, Baise City, Guangxi Zhuang Autonomous Region; and Zhangdong Village (ZD) in Hezhou City, Guangxi Zhuang Autonomous Region. These sites were selected because they represent important natural distribution areas of *O. microphylla* and encompass contrasting geographic habitats, including karst mountainous area, a plateau-to-plain transitional slope zone, and a hilly area. This sampling design allowed us to compare bacterial community patterns across plant compartments under different habitat contexts. Based on previous evidence that plant-associated bacterial communities are shaped by both host compartments and surrounding habitats, we hypothesized that: (i) bacterial communities of *O. microphylla* would show compartment-associated variation along the rhizosphere–root–stem–leaf continuum, with diversity decreasing from the rhizosphere to internal tissues; (ii) bacterial community composition would vary among geographic habitats, with rhizosphere communities showing stronger habitat-associated differences than endophytic communities; and (iii) some bacterial taxa would be repeatedly detected at relatively high abundance in internal tissues across habitats and could be considered candidate endophytic taxa for future validation. To test these hypotheses, we used high-throughput amplicon sequencing to characterize bacterial diversity, composition, candidate differential taxa, and co-occurrence patterns across rhizosphere soil, roots, stems, and leaves of *O. microphylla* from three contrasting geographic habitats. Specifically, this study aimed to: (1) characterize bacterial community patterns across different plant compartments and habitats; (2) identify candidate differential taxa associated with specific habitats or compartments; and (3) describe bacterial co-occurrence structures as statistical association patterns. This research provides a descriptive micro-ecological baseline for understanding bacterial community patterns associated with *O. microphylla* and identifies candidate taxa for future culture-dependent and functional studies.

## 2. Materials and Methods

### 2.1. Sample Collection

From November 2021 to January 2022, whole-plant continuum samples of *O. microphylla* were collected from three sites within its natural distribution range in Southwest China: Chazichong (CZC), Guizhou Province; Huangjingdong (HJD), Guangxi Zhuang Autonomous Region; and Zhangdong (ZD), Guangxi Zhuang Autonomous Region. These three sites were selected to represent contrasting landform types, including a karst mountainous area, a plateau-to-plain transitional slope zone, and a hilly area. At each site, candidate adult individuals were first identified in the core natural distribution area. Three healthy wild adult individuals were then randomly selected from these candidates, and sampled plants were spatially separated as far as possible to reduce spatial autocorrelation and minimize the influence of highly localized soil conditions. The selected plants had comparable growth status, with a height of 5–8 m and a diameter at breast height (DBH) of 8–10 cm, and showed no obvious symptoms of severe disease or abnormal growth. Rhizosphere soil, root, stem, and leaf samples were collected from each selected individual, properly labeled, and stored. Detailed sampling information is shown in Table 1.

Rhizosphere soil: Soil adhering to the root surface was collected from a 3 cm radius around the plant at a depth of 5–15 cm. Samples were placed in sterile zip-lock bags and labeled as CZC-rhizo (1, 2, 3), HJD-rhizo (1, 2, 3) and ZD-rhizo (1, 2, 3). Upon return to the laboratory, soil samples were passed through a 2-mm sieve and stored at −80 °C.

Root samples: Loose soil and debris attached to the root were gently brushed off after removing dead branches and large moss pieces. Five roots per plant were selected, and 1–2 cm segments were cut and stored in sterile bags, labeled as CZC-root (1, 2, 3), HJD-root (1, 2, 3) and ZD-root (1, 2, 3).

Stem and leaf samples: From each plant, 5–10 stems (1–2 cm segments) and leaves (5–10 pieces) were collected, placed in sterile bags and labeled accordingly (CZC-stem/leaf (1, 2, 3), HJD-stem/leaf (1, 2, 3), ZD-stem/leaf (1, 2, 3)).

### 2.2. Sample Surface Sterilization

On a clean bench, washed roots, stems, and leaves were cut into 1–2 cm segments. The segments were immersed in 75% ethanol for 3 min, rinsed 3–4 times with sterile water, immersed in 5% sodium hypochlorite for 3 min, and finally rinsed 5–8 times with sterile water. Excess water was removed, and samples were stored in 50 mL sterile centrifuge tubes. To verify the effectiveness of surface sterilization, aliquots of the final rinse water were plated on Luria–Bertani (LB) agar and incubated at 30 °C for 48 h. No visible microbial colonies were observed, indicating that surface-associated microorganisms had been effectively removed.

### 2.3. Instruments and Reagents

The E.Z.N.A.^®^ Soil DNA Kit (Omega Bio-tek, Norcross, GA, USA) was used for DNA extraction. PCR was performed using FastPfu Polymerase (TransGen Biotech, Beijing, China) and a T100 Thermal Cycler (BIO-RAD, Hercules, CA, USA). Electrophoresis was conducted using a JY600C instrument (Junyi Dongfang, Beijing, China). Illumina NextSeq2000 sequencing was performed by Shanghai Majorbio Bio-pharm Technology Co., Ltd (Shanghai, China).

### 2.4. DNA Extraction and Sequencing

Total genomic DNA was extracted following the E.Z.N.A.^®^ Soil DNA Kit protocol. DNA quality was checked via 1% agarose gel electrophoresis, and concentration/purity was measured using a NanoDrop 2000 (Thermo Scientific, Waltham, MA, USA). PCR amplification was performed using FastPfu Polymerase (TransGen Biotech, Beijing, China) and a T100 Thermal Cycler (BIO-RAD, Hercules, CA, USA). Library preparation and Illumina NextSeq2000 sequencing were performed by Shanghai Majorbio Bio-pharm Technology Co., Ltd.

Raw data were quality-controlled and merged using fastp (v0.19.6) and FLASH (v1.2.11), respectively. UPARSE (v7.1) was used for cluster operational taxonomic units (OTUs) at a 97% similarity threshold. Taxonomic classification was performed using the RDP classifier (v2.11) against the SILVA 16S rRNA database (v138). Although amplicon sequence variants (ASVs) provide finer taxonomic resolution, the OTU-based UPARSE pipeline was used to obtain a conservative overview of broad-scale bacterial community patterns across plant compartments and habitats. The limitations of this OTU-based strategy are acknowledged in the Section 4.

### 2.5. Co-Occurrence Network Construction

Bacterial co-occurrence networks were constructed using R. OTUs detected in at least 50% of samples within each group were retained for network construction. To mitigate compositional bias inherent in amplicon data, OTU abundance profiles were subjected to centered log-ratio (CLR) transformation before correlation analysis. Pairwise Spearman rank correlations were calculated using the psych package, and *p*-values were adjusted using the false discovery rate (FDR) method. Only strong and statistically significant correlations (|r| ≥ 0.6 and FDR-adjusted *p* < 0.05) were retained as network edges. Networks were constructed, topologically analyzed, and partitioned into modules using the igraph package in R, and visualized using Gephi (V0.10.1).

### 2.6. Statistical Analysis

All statistical analyses were performed using the Majorbio Cloud Platform and the R environment. Alpha diversity indices, including Chao1 and Shannon indices, were calculated using mothur (v1.9.1). Pairwise differences in alpha diversity between groups were tested using Wilcoxon rank-sum tests. Beta-diversity patterns were visualized using principal coordinate analysis (PCoA) based on Bray–Curtis distances. Differences in bacterial community composition among predefined groups were evaluated using analysis of similarities (ANOSIM). Candidate differential taxa among groups were identified using linear discriminant analysis effect size (LEfSe), with a linear discriminant analysis (LDA) score threshold > 2.5 and *p* < 0.05.

## 3. Results

### 3.1. Sequencing Depth and Bacterial Community Diversity

A total of 36 samples were obtained, representing three habitats, four plant compartments, and three biological replicates per compartment. These samples included rhizosphere soil (rhizo), root, stem and leaf samples from healthy *O. microphylla* individuals collected from Chazichong (CZC), Zhangdong (ZD) and Huangjingdong (HJD). Sequencing depth and OTU clustering information for each sample group are summarized in Table S1. Rarefaction curves based on the Shannon and Sobs indices gradually approached saturation with increasing sequencing depth (Figure S1), indicating that the sequencing depth was sufficient to capture the major bacterial diversity patterns and supporting subsequent community analyses.

**Figure 1:**
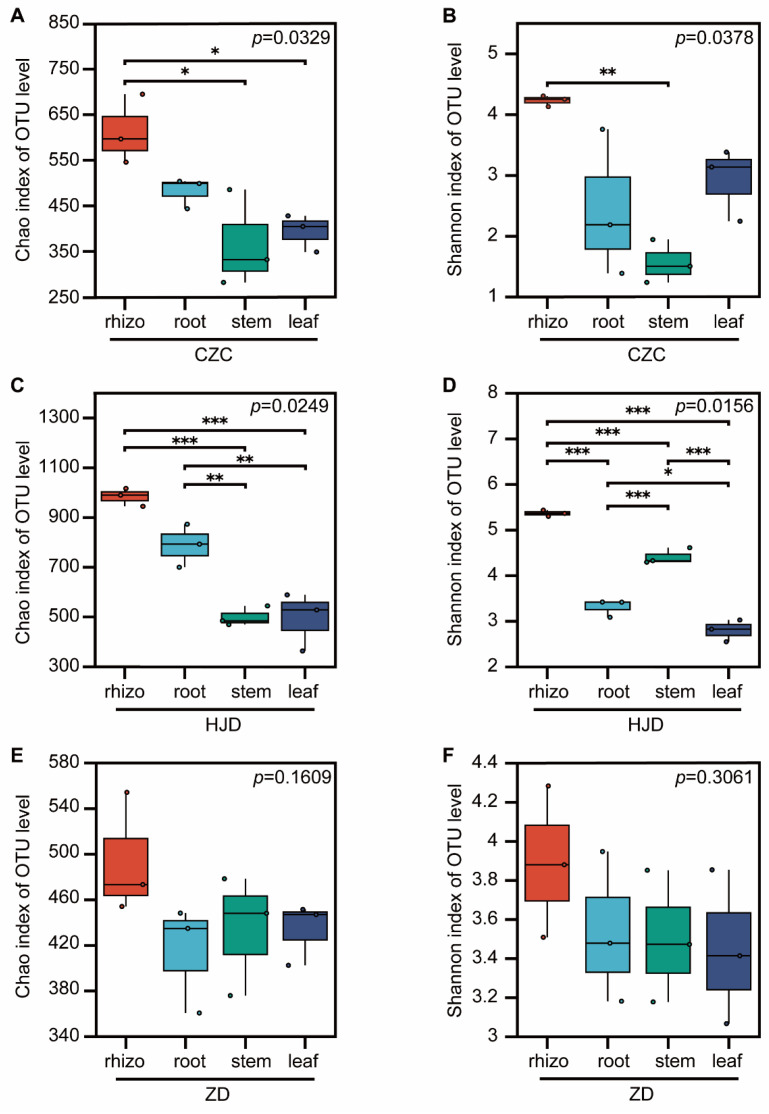
Alpha diversity of bacterial communities across the rhizosphere–root–stem–leaf continuum of *O. microphylla* in three habitats. Chao1 richness index in Chazichong (**A**), Huangjingdong (**C**), and Zhangdong (**E**). Shannon diversity index in Chazichong (**B**), Huangjingdong (**D**), and Zhangdong (**F**). CZC, Chazichong; HJD, Huangjingdong; ZD, Zhangdong; rhizo, rhizosphere soil. Boxes indicate the interquartile range, horizontal lines indicate medians, and points represent biological replicates (*n*= 3). Significance was tested using Wilcoxon rank-sum tests; * *p* < 0.05, ** *p* < 0.01, *** *p* < 0.001.

Alpha-diversity analysis revealed clear compartment-associated differences in bacterial richness and diversity across the whole-plant continuum (Figure 1). In all three habitats, rhizosphere samples generally showed higher Chao1 richness than internal tissues, while stems and leaves tended to exhibit lower richness. A similar pattern was observed for the Shannon index, although the strength of this trend varied among habitats. Among the three habitats, HJD generally showed higher bacterial richness across plant compartments, whereas ZD tended to show lower richness. Overall, these results indicate a compartment-associated decline in bacterial alpha diversity from the rhizosphere to internal tissues and suggest observable habitat-associated differences in bacterial diversity within this dataset.

### 3.2. Beta Diversity and Community Structure

**Figure 2:**
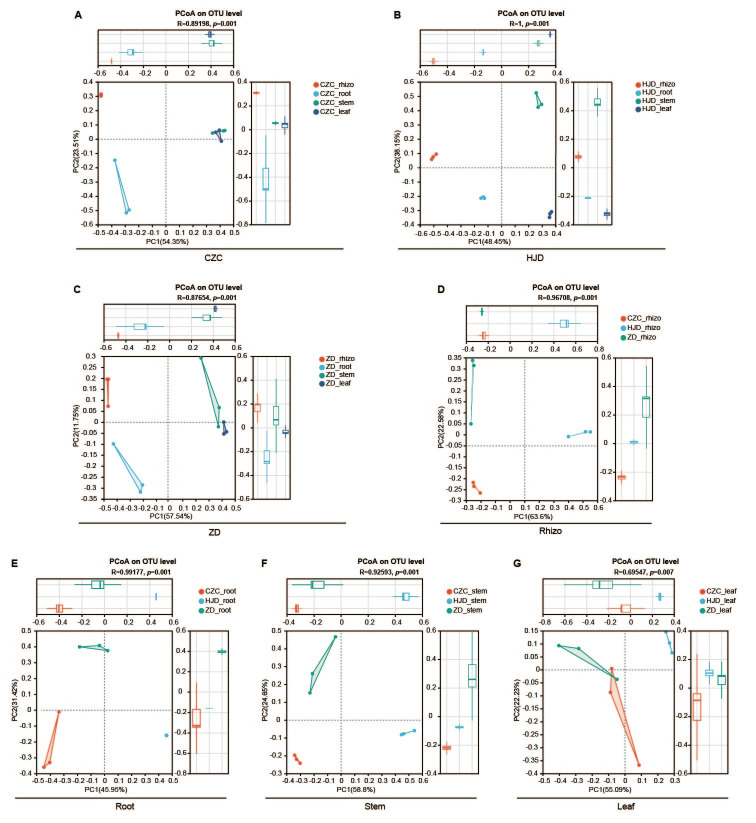
Beta-diversity analysis of bacterial communities across plant compartments and habitats of *O. microphylla* based on Bray–Curtis distances. Principal Coordinate Analysis (PCoA) was used to visualize differences in bacterial community composition. (**A**–**C**) PCoA ordinations comparing rhizosphere soil, root, stem, and leaf bacterial communities within each habitat: Chazichong (**A**), Huangjingdong (**B**), and Zhangdong (**C**). PCoA ordinations comparing bacterial communities from the same plant compartment among the three habitats: rhizosphere soil (**D**), root (**E**), stem (**F**), and leaf (**G**). Each point represents one biological replicate (*n* = 3 per group). Boxplots along the top and right margins show the distribution of samples along PC1 and PC2, respectively. PC1 and PC2 values indicate the percentage of variation explained by the first two principal coordinates. ANOSIM R and *p* values are shown above each ordination panel. CZC, Chazichong; HJD, Huangjingdong; ZD, Zhangdong; rhizo, rhizosphere soil.

To evaluate differences in bacterial community composition among plant compartments and habitats, beta-diversity analysis was performed using PCoA based on Bray–Curtis distances (Figure 2). Within each habitat, PCoA visualized clear compartment-associated grouping trends among rhizosphere, root, stem, and leaf samples. In HJD, the first two principal coordinates explained 48.45% and 38.15% of the total variation, respectively, and the observed compartment-associated separation was supported by ANOSIM (*p* = 0.001). Similar compartment-associated differences were observed in CZC (PC1 = 54.35%, PC2 = 23.51%; ANOSIM, *p* = 0.001) and ZD (PC1 = 57.54%, PC2 = 11.75%; ANOSIM, *p* = 0.001).

Cross-habitat comparisons within the same plant compartment further showed habitat-associated variation in bacterial community composition. Rhizosphere samples from CZC, HJD, and ZD showed clear grouping trends in the PCoA ordination, with the first two axes explaining 63.60% and 22.58% of the variation, respectively, and the difference was supported by ANOSIM (*p* = 0.001). Habitat-associated differences were also detected in root, stem, and leaf bacterial communities (*p* < 0.05), although the degree of separation varied among compartments. Overall, these results indicate that bacterial community composition in *O. microphylla* was associated with both plant compartment and geographic habitat, with PCoA providing ordination-based visualization and ANOSIM providing statistical support for group differences.

### 3.3. Community Composition and OTU Overlap

**Figure 3:**
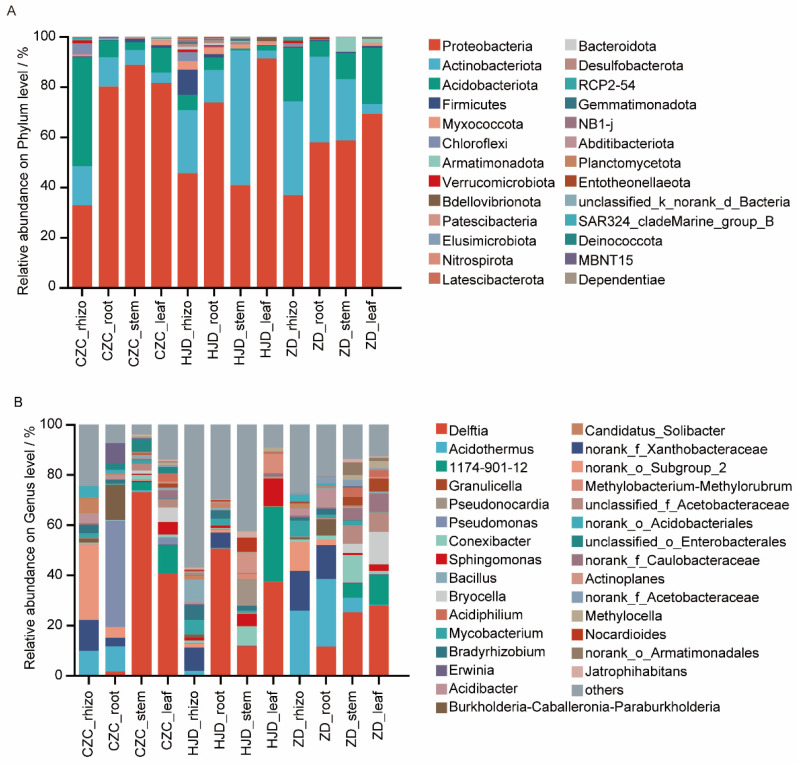
Taxonomic composition of bacterial communities across plant compartments and habitats of *O. microphylla*. (**A**) Relative abundance of dominant bacterial phyla. (**B**) Relative abundance of dominant bacterial genera. Low-abundance taxa were grouped as “Others.” CZC, Chazichong; HJD, Huangjingdong; ZD, Zhangdong; rhizo, rhizosphere soil.

Taxonomic composition analysis showed that dominant bacterial taxa varied across plant compartments and habitats (Figure 3; Table 2). At the phylum level, rhizosphere communities differed among habitats, with Acidobacteriota dominant in CZC, Proteobacteria dominant in HJD, and both Proteobacteria and Actinobacteriota dominant in ZD. In contrast, internal tissues across the three habitats were generally dominated by Proteobacteria, particularly in stem and leaf samples. At the genus level, dominant rhizosphere taxa also differed among habitats, including an unclassified genus of the order Subgroup 2 in CZC, an unclassified genus within the family Xanthobacteraceae in HJD, and *Acidothermus* in ZD. *Delftia* showed relatively high abundance in several endophytic compartments, especially in HJD roots, stems, and leaves. This pattern identifies *Delftia* as a candidate endophytic taxon of interest, but its ecological role cannot be inferred from the present amplicon data.

Shared and unique OTU patterns were further summarized using Venn diagrams (Figures S2 and S3). Within each habitat, OTUs shared among rhizosphere, root, stem, and leaf samples accounted for approximately 15–20% of the total OTUs, indicating that only a subset of bacterial taxa was common across the whole-plant continuum. Cross-habitat comparisons within the same plant compartment showed that shared OTUs among the three habitats accounted for approximately 15–30% of the total OTUs, while HJD showed the highest proportion of habitat-specific OTUs in each compartment. These results indicate distinct OTU distribution patterns across plant compartments and habitats, but they should be interpreted as descriptive summaries of OTU overlap rather than evidence of transmission barriers or ecological isolation.

### 3.4. Candidate Differential Taxa Identified by LEfSe

**Figure 4:**
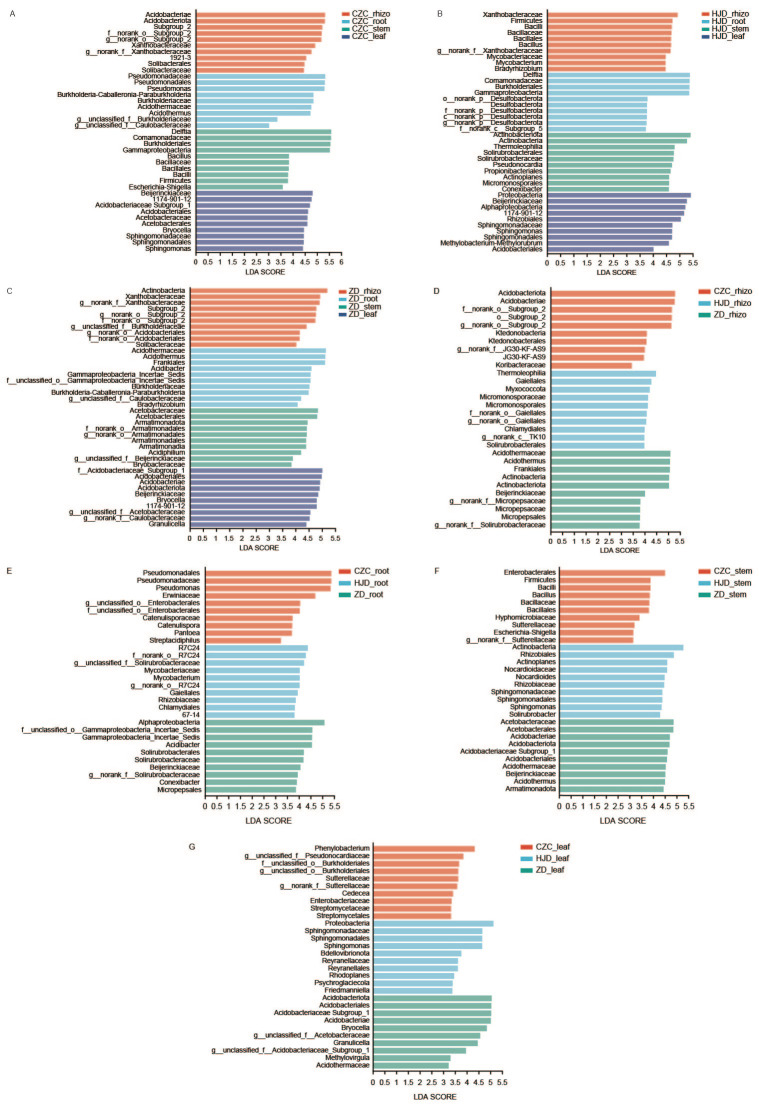
Candidate differential taxa associated with plant compartments and habitats of *O. microphylla* identified by LEfSe analysis. (**A**–**C**) LDA comparing rhizosphere soil, root, stem, and leaf bacterial communities within each habitat: Chazichong (**A**), Huangjingdong (**B**), and Zhangdong (**C**). (**D**–**G**) LDA comparing comparing bacterial communities from the same plant compartment among the three habitats: rhizosphere soil (**D**), root (**E**), stem (**F**), and leaf (**G**). Taxa with LDA scores > 2.5 and *p* < 0.05 are shown. Colors indicate the plant compartment or habitat in which each taxon showed higher relative abundance. LEfSe, Linear Discriminant Analysis Effect Size; LDA, Linear Discriminant Analysis; CZC, Chazichong; HJD, Huangjingdong; ZD, Zhangdong; rhizo, rhizosphere soil.

LEfSe analysis was performed to identify candidate differential taxa associated with plant compartments and habitats (LDA score > 2.5, *p* < 0.05; Figure 4). Within each habitat, different plant compartments showed distinct sets of candidate differential taxa. In CZC, candidate taxa were mainly associated with the rhizosphere and included members of the Xanthobacteraceae family and an unclassified genus of the Subgroup 2 order, whereas *Pseudomonas* and *Acidothermus* were more strongly associated with roots, *Delftia* and *Bacillus* with stems, and *Bryocella* and *Sphingomonas* with leaves. In HJD, *Bacillus* and an unclassified genus of Xanthobacteraceae were associated with the rhizosphere, *Delftia* with roots, *Pseudonocardia*, *Actinoplanes*, and *Conexibacter* with stems, and *Sphingomonas* and *Methylobacterium* with leaves. In ZD, candidate differential taxa included unclassified members of the Subgroup 2 order and Burkholderiaceae/Xanthobacteraceae-related taxa in the rhizosphere, *Acidibacter*, *Acidothermus*, *Burkholderia*-*Caballeronia*-*Paraburkholderia*, and *Bradyrhizobium* in roots, *Acidibacter* and Burkholderiaceae-related taxa in stems, and Acetobacteraceae-, Caulobacteraceae-, and *Granulicella*-related taxa in leaves.

Comparisons of the same plant compartment across habitats also revealed habitat-associated candidate differential taxa. For rhizosphere samples, CZC was characterized by an unclassified genus of the Subgroup 2 order and an unclassified genus from the JG30-KF-AS9 family, HJD by unclassified genera from the Gaiellales order and TK10 class, and ZD by *Acidothermus* and unclassified genera from the Micropepsaceae and Solirubrobacteraceae families. For root samples, *Pseudomonas*, *Streptomyces*, *Pantoea*, and *Streptacidiphilus* were associated with CZC, *Mycobacterium* and Solirubrobacteraceae-related taxa with HJD, and *Acidibacter*, *Conexibacter*, and Solirubrobacteraceae-related taxa with ZD. Habitat-associated candidate taxa were also observed in stem and leaf samples.

Overall, the LEfSe results indicate that bacterial taxa with differential relative abundance varied across both plant compartments and habitats. These taxa should be interpreted as candidate differential taxa identified from amplicon-based relative-abundance data, rather than as validated functional biomarkers.

### 3.5. Bacterial Co-Occurrence Network Patterns

**Figure 5:**
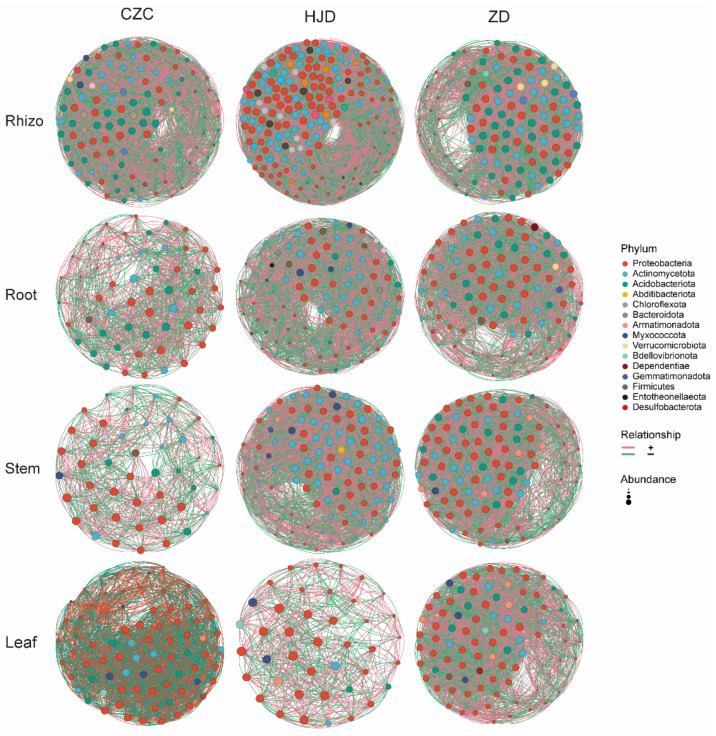
Bacterial co-occurrence network patterns across plant compartments and habitats of *O. microphylla*. Networks were constructed based on Spearman correlations after centered log-ratio (CLR) transformation of OTU abundance profiles. Only strong and statistically significant correlations were retained as network edges (|r| ≥ 0.6 and FDR-adjusted *p* < 0.05). Nodes represent bacterial OTUs, and node colors indicate bacterial phyla. Edge colors indicate positive and negative statistical correlations. Node size represents relative abundance. CZC, Chazichong; HJD, Huangjingdong; ZD, Zhangdong; rhizo, rhizosphere soil; OTU, operational taxonomic unit; FDR, false discovery rate. Network edges represent statistical co-occurrence associations and should not be interpreted as direct biological interactions.

Bacterial co-occurrence networks were constructed for different plant compartments and habitats of *O. microphylla* to describe statistical association patterns among bacterial OTUs (Figure 5). Network structures varied across habitats and plant compartments. Among rhizosphere networks, HJD showed the largest network size and highest complexity, with 198 nodes, 12,352 edges, and an average degree of 124.768 (Figure S5). In comparison, the average degrees of the CZC and ZD rhizosphere networks were 71.621 and 79.158, respectively. Across the networks, both positive and negative correlations were observed, with positive correlations accounting for approximately half of the total edges (Figure S5). These patterns indicate habitat- and compartment-associated differences in bacterial co-occurrence structure, but they should be interpreted as statistical associations rather than direct biological interactions.

Network degree distributions also differed among habitats and plant compartments (Figure S5). The degrees of CZC root and stem networks were significantly lower than those of HJD and ZD (*p* < 0.001), and leaf networks also showed significant differences among the three habitats (*p* < 0.001). Genera with high degree values were identified as topologically prominent nodes within the corresponding networks. The top 10 genera ranked by degree had the highest number of edges in the HJD rhizosphere network (1434 edges), whereas the HJD leaf network contained 238 edges among the top 10 genera, slightly more than the CZC stem network (234 edges). These degree-based patterns highlight taxa that are structurally prominent in the statistical networks, but they do not imply verified ecological functions or direct roles in community stability.

## 4. Discussion

### 4.1. Compartment-Associated Bacterial Diversity and Community Structure

This study revealed clear compartment-associated bacterial community patterns across the rhizosphere–root–stem–leaf continuum of *O. microphylla*. Alpha-diversity analysis showed that bacterial richness and diversity were generally higher in the rhizosphere than in internal tissues, indicating a decline in bacterial alpha diversity from the external soil-associated compartment to endophytic compartments. Similar compartment-associated decreases in microbial diversity have been reported in other plant systems, including woody plants, where the rhizosphere usually harbors a larger microbial pool than internal tissues because it is directly connected to the surrounding soil environment and root exudates [15,25,26,27]. Studies on field-grown woody plants, such as poplar, have also shown that rhizosphere, root, stem, and leaf compartments can harbor distinct bacterial communities [28]. This pattern is consistent with compartment-associated structuring, but it should not be interpreted as direct proof of a niche-filtering mechanism. Our study did not directly quantify ecological assembly processes such as selection, drift, dispersal limitation, or microbial source tracking. Therefore, the observed diversity gradient should be understood as a descriptive community pattern. This distinction is important because plant-associated microbiomes are shaped by multiple interacting factors, including host traits, plant compartment, local species pools, and environmental context [15].

Beta-diversity analysis further showed that bacterial community composition was associated with plant compartment. PCoA ordination showed compartment-associated grouping trends within each habitat, and ANOSIM provided statistical support for compositional differences among compartments. OTU overlap analysis also indicated that only a subset of OTUs was shared across the whole-plant continuum. These Venn diagram results provide useful descriptive summaries of shared and unique OTUs, but they do not demonstrate transmission barriers, microbial movement, or ecological isolation. Taken together, the alpha-diversity, beta-diversity, taxonomic composition, and OTU overlap results indicate clear compartment-associated bacterial community patterns in *O. microphylla*.

At the taxonomic level, internal tissues were generally dominated by Proteobacteria, especially in stem and leaf samples. Proteobacteria are frequently reported as abundant endophytic bacteria in many plant species, and some members of this phylum have been associated with plant colonization and adaptation to internal plant environments [25,26,28]. However, the present amplicon data only indicate relative-abundance patterns and cannot determine the physiological mechanisms underlying this enrichment. Therefore, the dominance of Proteobacteria in internal tissues should be viewed as a compositional feature of the *O. microphylla* microbiome that requires future functional investigation.

### 4.2. Habitat-Associated Variation in Bacterial Communities

In addition to compartment-associated differences, bacterial communities also varied among the three geographic habitats. Rhizosphere bacterial communities showed particularly clear habitat-associated differentiation, while root, stem, and leaf communities also displayed habitat-associated variation to varying degrees. This result suggests that bacterial communities associated with *O. microphylla* are related to both plant compartments and local habitat context. Such dual effects of host compartment and environmental background have been widely reported in plant microbiome studies, where external compartments such as the rhizosphere are often more directly exposed to local soil conditions, whereas endophytic compartments may be shaped by both host-associated and environmental factors [15,28]. Similar habitat-associated rhizosphere bacterial variation has also been reported in Pinus squamata, a plant species with extremely small populations, across different conservation sites [21].

The higher bacterial richness observed in HJD across several compartments may reflect habitat-associated differences in the available microbial species pool. However, because matched soil physicochemical properties were not measured in this study, the environmental factors underlying these habitat-associated differences cannot be directly determined. Landform type, soil pH, nutrient status, moisture, and microclimatic conditions may all contribute to bacterial community variation, but these remain hypotheses that require direct testing. For example, the enrichment of Acidobacteriota-related candidate taxa in ZD may be consistent with previous reports that red or lateritic red soils in parts of Guangxi are often acidic [29]. However, this interpretation should be considered tentative because soil pH was not measured in the present samples.

LEfSe analysis identified candidate differential taxa associated with specific plant compartments and habitats. These taxa provide useful statistical indicators of relative-abundance differences, but they should not be interpreted as validated functional biomarkers. Given the small sample size and lack of independent validation, the LEfSe results are best viewed as a hypothesis-generating resource for selecting taxa of interest in future culture-dependent and functional studies. This cautious interpretation is particularly important for endangered plant systems, where field sampling is limited and microbial functions cannot be inferred from relative abundance alone.

### 4.3. Candidate Endophytic Taxa and Co-Occurrence Network Patterns

Among the genera detected in this study, *Delftia* showed relatively high abundance in several endophytic compartments, especially in HJD roots, stems, and leaves. This pattern suggests that *Delftia* can be considered a candidate endophytic taxon of interest in *O. microphylla*, rather than a validated functional member or central driver of the observed community patterns. Previous studies have reported that some *Delftia* strains can promote plant growth [30,31] and delftibactin produced by *Delftia* has been associated with metal chelation and iron-related processes [32,33]. These studies provide useful literature context, but they should not be taken as evidence for the function of *Delftia* in *O. microphylla*. Because our study did not isolate *Delftia* strains, test their colonization ability, or evaluate their effects on *O. microphylla* growth or stress tolerance, the potential ecological role of *Delftia* in this host remains speculative and requires culture-dependent isolation, genome-based characterization, and inoculation experiments.

The co-occurrence network analysis revealed habitat- and compartment-associated differences in bacterial network structure. HJD rhizosphere samples showed the largest network size and highest average degree among rhizosphere networks, suggesting a more complex statistical co-occurrence structure in this habitat. However, correlation-based networks do not demonstrate direct biological interactions. Positive correlations may reflect shared habitat preferences, similar responses to unmeasured environmental factors, or potential cooperative relationships, whereas negative correlations may reflect divergent niche preferences, indirect associations, or other unmeasured factors. Similarly, genera with high degree values should be interpreted as topologically prominent nodes within statistical networks, not as confirmed keystone taxa or functionally important organisms. This interpretation is consistent with recent cautions that microbial co-occurrence networks are best used as hypothesis-generating tools, especially when based on compositional amplicon data [34,35,36].

### 4.4. Conservation Implications, Limitations, and Future Prospects

For endangered plants, microbiome surveys can provide a useful micro-ecological baseline for understanding plant-associated microbial community patterns and identifying candidate microbial taxa for future validation. Recent work on the endangered *L. tsingtauense*, for example, showed that microbiome-guided isolation of native rhizosphere fungi identified *T. longibrachiatum*, which colonized host roots and promoted root growth under experimental conditions [24]. This example illustrates that descriptive microbiome surveys can serve as an initial step toward microbial resource discovery, but only when followed by isolation, inoculation, compatibility testing, and functional validation.

In the present study, the identification of candidate endophytic taxa and habitat-associated candidate differential taxa provides a preliminary list of bacterial taxa for future research on *O. microphylla*. Studies on synthetic microbial communities (SynComs) in other plant systems provide useful conceptual background for future microbiome-based studies [37,38,39]. However, the present dataset does not provide application-ready evidence for microbiome-assisted conservation. Any future conservation-oriented application would first require the isolation of candidate strains, confirmation of plant-growth-promoting or stress-resistance traits, evaluation of compatibility among taxa, and in planta validation. Therefore, SynComs or other microbiome-based interventions should be considered only as possible future research directions rather than outcomes or recommendations of this study.

Several limitations should be acknowledged. First, only three adult individuals were sampled at each site. This limited sample size reflects the rarity and protected status of *O. microphylla*, but it constrains the statistical power and generalizability of habitat-level conclusions. Future studies should include larger sample sizes, more populations, and repeated seasonal sampling where permitted. Second, matched soil physicochemical data were not collected. As a result, the environmental drivers of habitat-associated bacterial community variation cannot be directly identified. Future work should integrate microbiome profiling with soil pH, nutrient availability, organic matter, moisture, and microclimatic measurements. Third, this study used 97% OTU clustering rather than ASV-based inference. Although this approach is sufficient for describing broad-scale community patterns, it limits fine-scale taxonomic resolution. Future studies using ASV-based pipelines, metagenomics, metatranscriptomics, and culture-dependent approaches will be needed to refine taxonomic resolution and link candidate taxa to ecological functions. Finally, because 16S rRNA amplicon sequencing provides relative-abundance data, it cannot establish microbial functions, host benefits, or causal ecological mechanisms. Functional validation through strain isolation, genome analysis, inoculation assays, and field trials will be essential to determine whether candidate bacteria contribute to the growth, regeneration, or stress tolerance of *O. microphylla*.

Overall, this study provides a descriptive micro-ecological baseline for understanding bacterial community patterns across the whole-plant continuum of *O. microphylla*. The results show that bacterial communities are associated with both plant compartment and geographic habitat, and identify candidate taxa that may be examined in future culture-dependent studies. These findings should be interpreted as descriptive community patterns rather than mechanistic or application-ready evidence.

## 5. Conclusions

This study characterized bacterial community patterns across the rhizosphere–root–stem–leaf continuum of *O. microphylla* in three geographic habitats. The results showed that bacterial alpha diversity generally declined from the rhizosphere to internal tissues and that bacterial community composition differed by both plant compartment and habitat. Rhizosphere communities showed habitat-associated differences, whereas internal tissues were generally dominated by Proteobacteria. At the genus level, *Delftia* was among the taxa showing relatively high abundance in several endophytic compartments and may be considered a candidate endophytic taxon of interest for future validation.

Overall, this study provides a descriptive micro-ecological baseline for understanding plant-associated bacterial communities of *O. microphylla*. The present 16S rRNA amplicon data do not establish microbial functions, host benefits, causal ecological processes, or application-ready conservation evidence. Future studies should integrate larger-scale sampling, soil physicochemical measurements, strain isolation, metagenomic or metatranscriptomic analyses, and inoculation experiments to evaluate the ecological roles of candidate taxa.

## Figures and Tables

**Table 1:** Sample collection information.

Sample ID	Collection Sample		Collection Site	Longitude and Latitude	Landform
CZC	*O. microphylla*	Rhizosphere	Taijiang County, Qiandongnan Miao and Dong Autonomous Prefecture, Guizhou Province	26°12′3.08″ N, 108°47′46.01″ E	Karst (limestone) mountainous area
		Root			
		Stem			
		Leaf			
HJD		Rhizosphere	Longlin Various Nationalities Autonomous County, Baise City, Guangxi Zhuang Autonomous Region	24°48′29.66″ N, 106°22′29.16″ E	A transitional slope zone from the plateau to the hills and plains
		Root			
		Stem			
		Leaf			
ZD		Rhizosphere	Hezhou City, Guangxi Zhuang Autonomous Region	23°58′45.82″ N, 111°14′0.20″ E	Hilly land
		Root			
		Stem			
		Leaf			

**Table 2:** Statistics of bacterial community taxonomic groups.

Taxonomy Sample	Phylum	Class	Order	Family	Genus	Species
CZC_rhizo	22	38	87	132	214	335
CZC_root	21	33	80	116	179	281
CZC_stem	18	30	68	101	174	272
CZC_leaf	17	30	67	102	162	247
HJD_rhizo	23	51	117	184	316	582
HJD_root	24	52	116	179	305	535
HJD_stem	20	32	74	112	197	296
HJD_leaf	20	43	90	143	244	387
ZD_rhizo	19	32	77	110	165	266
ZD_root	16	29	71	103	162	255
ZD_stem	17	28	69	95	158	237
ZD_leaf	14	27	69	93	148	227

## Data Availability

The original data presented in the study are openly available in NCBI Sequence Read Archive (SRA) database at [https://dataview.ncbi.nlm.nih.gov/object/PRJNA1435581?reviewer=2fpqt9pu65pi10o3dh593l2me4], reference number [PRJNA1435581].

## Supplementary Materials

The following supporting information can be downloaded at: https://www.mdpi.com/article/10.3390/microorganisms14051143/s1. Table S1: Sequencing depth and OTU clustering summary for each sample group. Figure S1: Rarefaction curves of bacterial communities across rhizosphere, root, stem, and leaf samples of *O. microphylla* from three habitats. (A) Shannon index rarefaction curves; (B) Sobs index rarefaction curves. CZC, Chazichong; HJD, Huangjingdong; ZD, Zhangdong; rhizo, rhizosphere soil. Figure S2: Shared and unique OTU patterns among plant compartments within each habitat of *O. microphylla*. Venn diagrams show shared and unique OTUs among rhizosphere soil, root, stem, and leaf samples in (A) Chazichong (CZC), (B) Huangjingdong (HJD), and (C) Zhangdong (ZD). Numbers indicate OTU counts, and percentages indicate the proportion of OTUs in each region relative to the total OTUs included in the corresponding comparison. CZC, Chazichong; HJD, Huangjingdong; ZD, Zhangdong; rhizo, rhizosphere soil; OTU, operational taxonomic unit. Figure S3: Shared and unique OTU patterns among habitats within the same plant compartment of *O. microphylla*. Venn diagrams show shared and unique OTUs among Chazichong (CZC), Huangjingdong (HJD), and Zhangdong (ZD) for rhizosphere soil (A), root (B), stem (C), and leaf (D) samples. Numbers indicate OTU counts, and percentages indicate the proportion of OTUs in each region relative to the total OTUs included in the corresponding comparison. OTU, operational taxonomic unit. Figure S4: LEfSe cladograms showing candidate differential taxa associated with plant compartments and habitats of *O. microphylla*. (A–C) Cladograms comparing rhizosphere soil, root, stem, and leaf bacterial communities within each habitat: Chazichong (A), Huangjingdong (B), and Zhangdong (C). (D–G) Cladograms comparing bacterial communities from the same plant compartment among the three habitats: rhizosphere soil (D), root (E), stem (F), and leaf (G). Different colors indicate the plant compartment or habitat in which the corresponding taxa showed higher relative abundance, whereas yellow nodes indicate taxa with no significant difference among groups. Only taxa meeting the LEfSe threshold of LDA score > 2.5 and *p* < 0.05 are shown. LEfSe, Linear Discriminant Analysis Effect Size; LDA, Linear Discriminant Analysis; CZC, Chazichong; HJD, Huangjingdong; ZD, Zhangdong; rhizo, rhizosphere soil. Figure S5: Topological properties of bacterial co-occurrence networks across plant compartments and habitats of *O. microphylla*. (A) Number of nodes in each network. (B) Degree distribution of bacterial OTUs across networks. (C) Number of positive and negative edges in the full co-occurrence networks. (D) Number of positive and negative edges among the top 10 genera ranked by degree in each network. Positive and negative edges represent statistically significant positive and negative Spearman correlations, respectively, after centered log-ratio (CLR) transformation and false discovery rate (FDR) correction (|r| ≥ 0.6 and FDR-adjusted *p* < 0.05). CZC, Chazichong; HJD, Huangjingdong; ZD, Zhangdong; rhizo, rhizosphere soil; OTU, operational taxonomic unit.

## Abbreviations

The following abbreviations are used in this manuscript:

CZC	Samples collected from Taijiang County, Qiandongnan Miao and Dong Autonomous Prefecture, Guizhou Province
HJD	Samples collected from Longlin Various Nationalities Autonomous County, Baise City, Guangxi Zhuang Autonomous Region
ZD	Samples collected from Hezhou City, Guangxi Zhuang Autonomous Region
DBH	diameter at breast height
ISR	Induced Systemic Resistance
PGPB	plant growth-promoting bacteria
ACC	1-aminocyclopropane-1-carboxylate
PCoA	Principal Coordinate Analysis
ANOSIM	Analysis of similarities
LEfSe	Linear Discriminant Analysis Effect Size
LDA	Linear Discriminant Analysis
OTU	Operational taxonomic unit
SynCom/SynComs	Synthetic Microbial Community/Synthetic Microbial Communities
