# Supplementary figures and images for: Bacterial Community Patterns Across the Whole-Plant Continuum of Ormosia microphylla in Diverse Habitats
Source: Microorganisms. 2026 May 19;14(5):1143. doi: 10.3390/microorganisms14051143 (PMC13209703; doi:10.3390/microorganisms14051143)

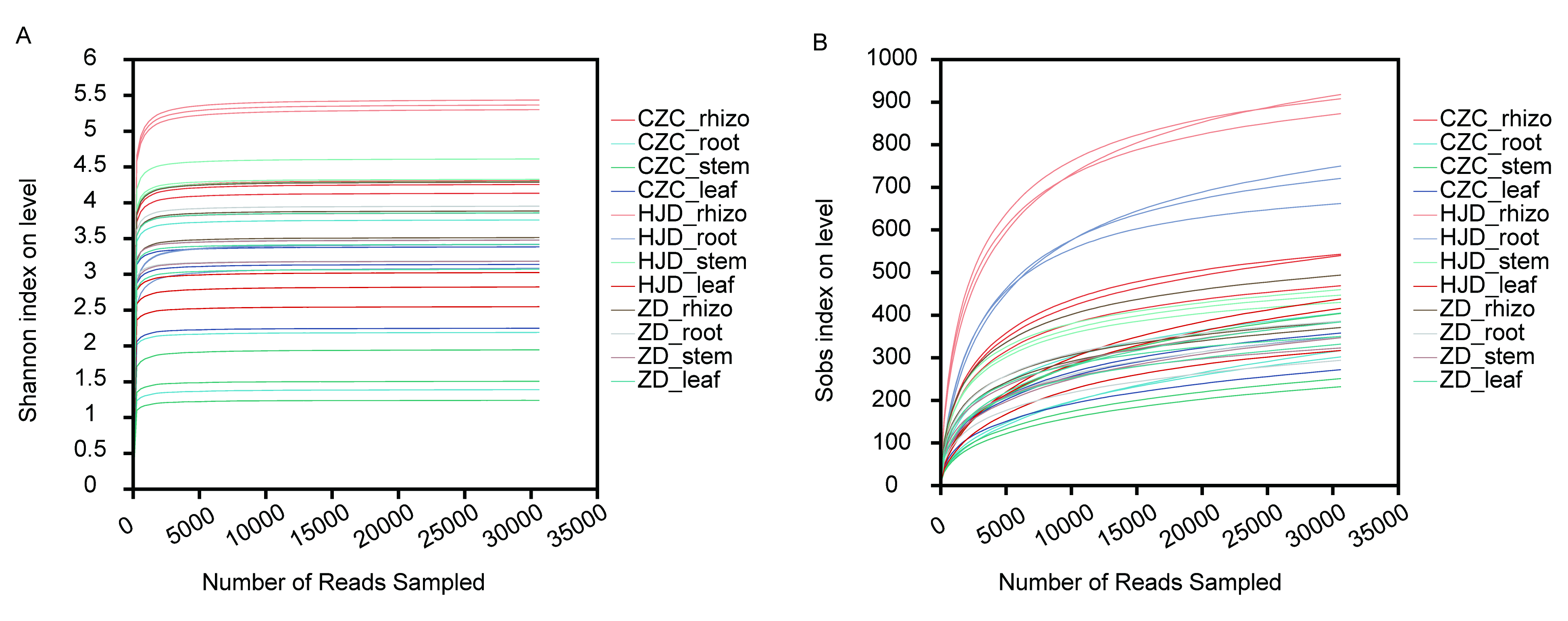

Supplement: Supplementary file 1 [file microorganisms-14-01143-s001.zip › FigureS1.tif]

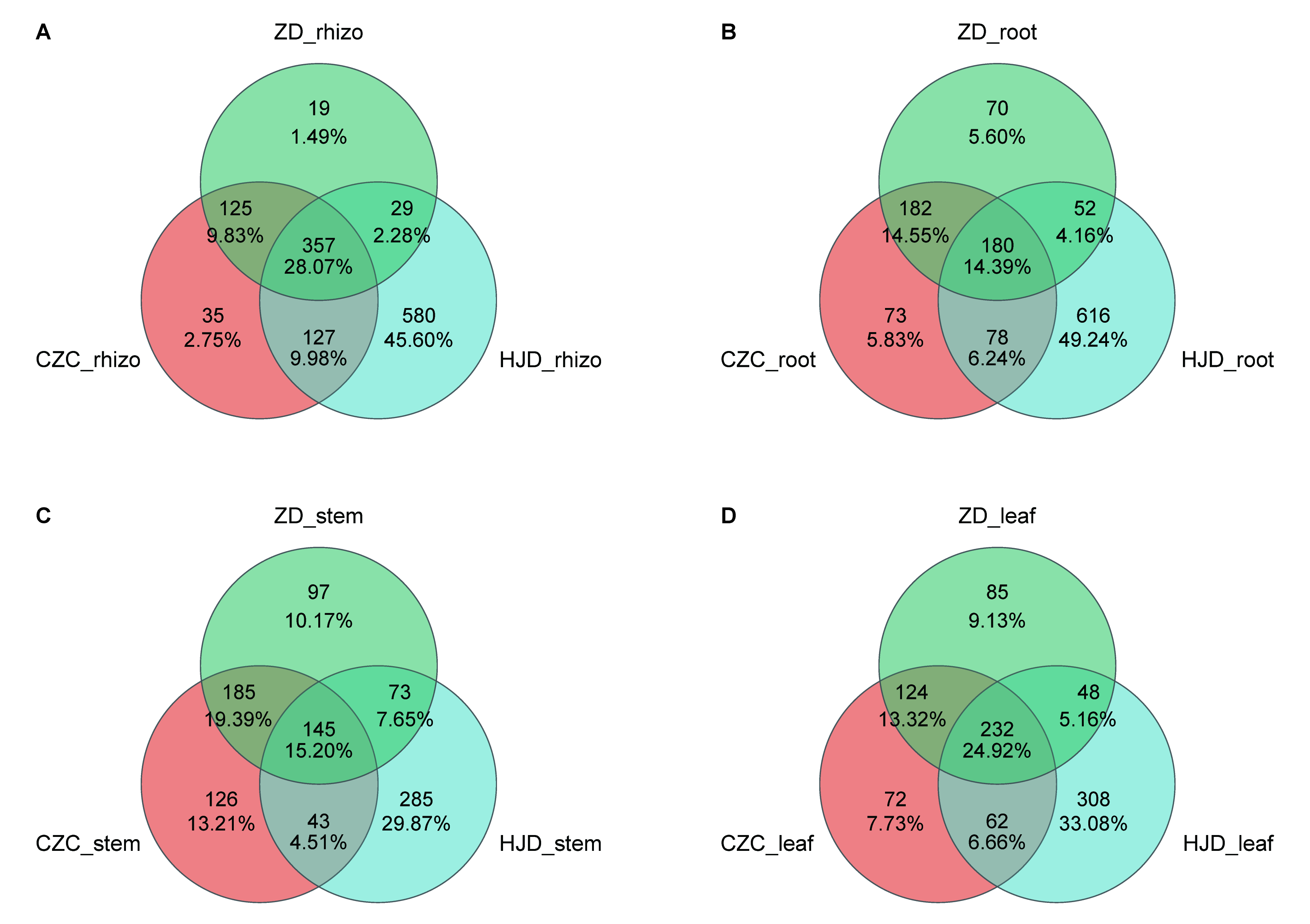

Supplement: Supplementary file 1 [file microorganisms-14-01143-s001.zip › FigureS2.tif]

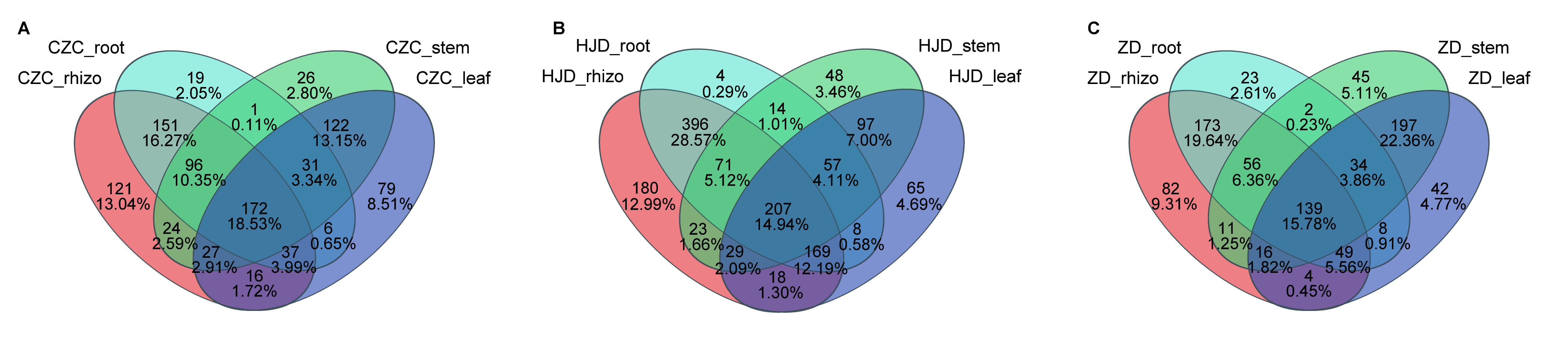

Supplement: Supplementary file 1 [file microorganisms-14-01143-s001.zip › FigureS3.tif]

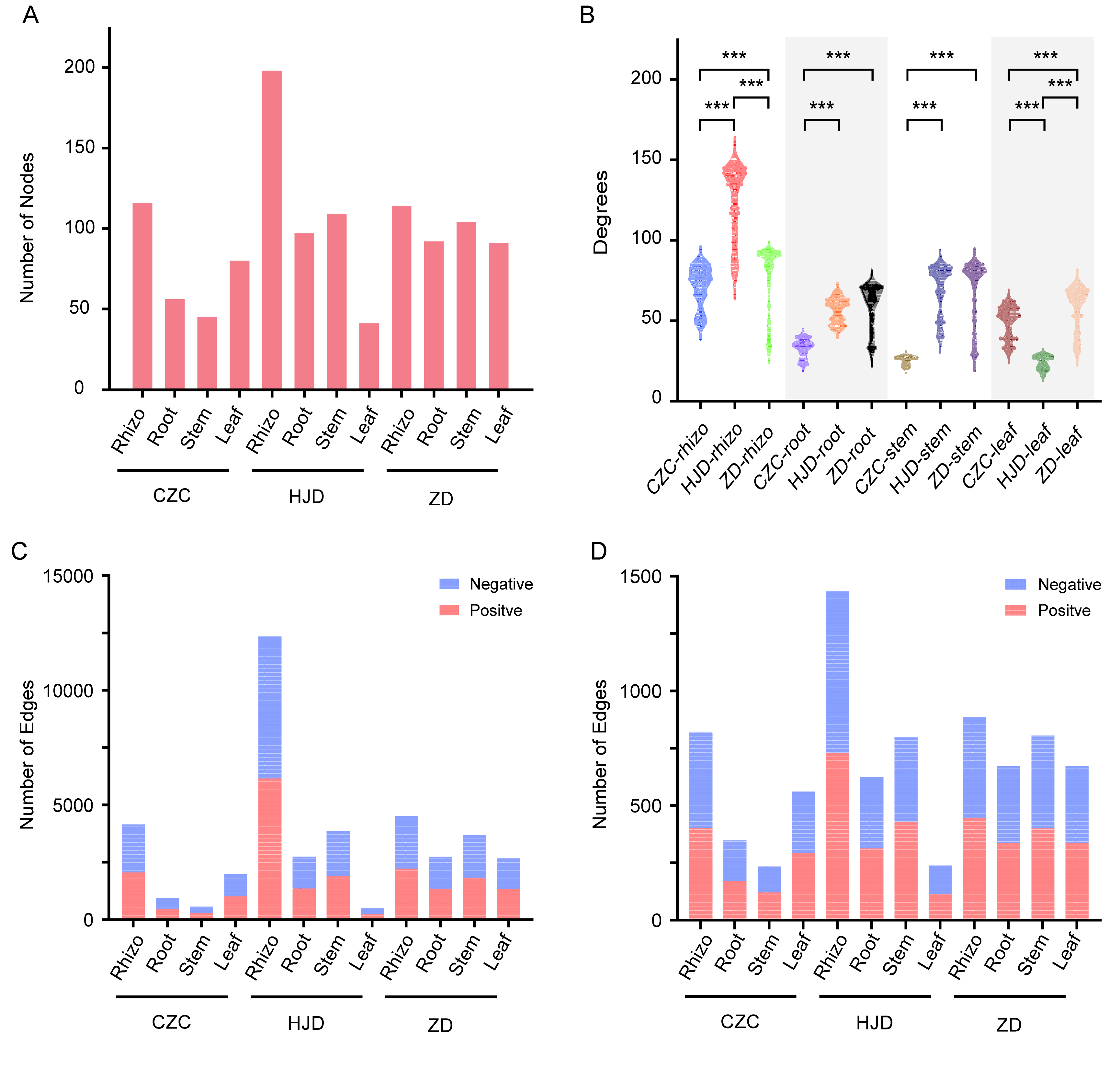

Supplement: Supplementary file 1 [file microorganisms-14-01143-s001.zip › FigureS5.tif]
